# Causes and consequences of acidification in the Baltic Sea: implications for monitoring and management

**DOI:** 10.1038/s41598-023-43596-8

**Published:** 2023-09-28

**Authors:** Erik Gustafsson, Jacob Carstensen, Vivi Fleming, Bo G. Gustafsson, Laura Hoikkala, Gregor Rehder

**Affiliations:** 1 Baltic Nest Institute, Baltic Sea Centre, Stockholm University, Stockholm, Sweden; 2 Department of Ecoscience, Aarhus University, Roskilde, Denmark; 3 Marine and Freshwater Solutions, Finnish Environment Institute, Helsinki, Finland; 4 Tvärminne Zoological Station, University of Helsinki, Hanko, Finland; 5 Leibniz Institute for Baltic Sea Research Warnemünde (IOW), Rostock, Germany

**Keywords:** Carbon cycle, Climate-change ecology, Ocean sciences, Climate and Earth system modelling, Environmental health

## Abstract

Increasing atmospheric CO_2_ drives ocean acidification globally. In coastal seas, acidification trends can however be either counteracted or enhanced by other processes. Ecosystem effects of acidification are so far small in the Baltic Sea, but changes should be anticipated unless CO_2_ emissions are curbed. Possible future acidification trends in the Baltic Sea, conditional on CO_2_ emissions, climate change, and changes in productivity, can be assessed by means of model simulations. There are uncertainties regarding potential consequences for marine organisms, partly because of difficulties to assign critical thresholds, but also because of knowledge gaps regarding species’ capacity to adapt. Increased temporal and spatial monitoring of inorganic carbon system parameters would allow a better understanding of current acidification trends and also improve the capacity to predict possible future changes. An additional benefit is that such measurements also provide quantitative estimates of productivity. The technology required for precise measurements of the inorganic carbon system is readily available today. Regularly updated status evaluations of acidification, and the inorganic carbon system in general, would support management when assessing climate change effects, eutrophication or characteristics of the pelagic habitats. This would, however, have to be based on a spatially and temporally sufficient monitoring program.

## Introduction

Present-day anthropogenic CO_2_ emissions amount to some 40 billion tons of CO_2_ per year^1^. Almost half of this CO_2_ accumulates in the atmosphere, currently leading to an annual rise of the atmospheric CO_2_ partial pressure (pCO_2_) by approximately 2 µatm. Surface water pCO_2_ tends to equilibrate with atmospheric pCO_2_, leading to an enhanced aquatic CO_2_ concentration^2^, which causes pH to decrease. The pH decrease associated with enhanced CO_2_ concentrations is predictable in surface waters of the open ocean, because other processes that cause long-term pH trends tend to be slow compared to the CO_2_ effect. The CO_2_ uptake by oceans presently amounts to about a quarter of the anthropogenic emissions^1^, resulting in a pH decrease of approximately 0.02 per decade, as well as a gradual decline in saturation levels of calcium carbonate minerals^3^. If anthropogenic CO_2_ emissions are not controlled, the atmospheric pCO_2_ might in the worst-case scenario increase from present-day ~ 420 µatm to 950 µatm by year 2100^4^, decreasing pH in ocean surface waters by 0.3 units on average^5^.

In contrast to the open ocean, uniform long-term decreasing pH trends are not generally observed in coastal seas^6–10^. Because of the more direct links between terrestrial and marine reservoirs of carbon and nutrients in coastal seas, the acidification driven by increasing atmospheric pCO_2_ can be either enhanced or counteracted by changes in runoff and salinity, changes in nutrient loads, productivity, and oxygen conditions, and also changes in rock weathering and riverine supply of total alkalinity (A_T_).

Baltic Sea water is a mixture of ocean water and freshwater. The proportions of ocean water and freshwater vary, both depending on location and over time. Riverine A_T_ is usually lower than oceanic A_T_, which implies that the lower salinity in the Baltic Sea is typically associated with A_T_ below oceanic values. There are, however, exceptions. Rivers draining the limestone-dominated south-eastern catchment areas of the Baltic Sea can have A_T_ concentrations that exceed the typical North Sea A_T_ (~ 2250 µmol kg^−1^) considerably^11–13^. For example, mixing with freshwater sources in the Gulf of Riga would produce higher A_T_ in that particular sub-basin where riverine A_T_ can reach 3000 µmol kg^−1^—approximately two times higher than Baltic Sea mean A_T_ (~ 1530 µmol kg^−1^)^14^. Changes in rock weathering can modify riverine A_T_ in addition to effects caused by dilution or increased A_T_ concentration by either enhanced or reduced water flow.

In high-latitude marine systems such as the Baltic Sea, productivity patterns are characterized by strong seasonal variations and a seasonal decoupling between production and respiration^9^. Algal blooms in spring and summer temporarily increase surface water pH in the Baltic Sea because of CO_2_ uptake by the autotrophs. CO_2_ is again released, and pH decreases, as the organic material decomposes during late summer and autumn. In stratified water bodies there can furthermore be a spatial decoupling between production and respiration. Eutrophication can further augment pH in spring and summer, but can also generate a local increasing pH trend over time if the produced organic material is subsequently exported to the deep water or an adjacent basin. However, eutrophication can at the same time enhance acidification in sub-surface water by decomposition of excess organic matter^15, 16^.

Analyses of long-term A_T_ trends in the Baltic Sea indicate that ocean acidification has been partly counteracted by other processes in the central and northern sub-basins^7^. There are at the same time contrasting examples from Danish estuaries where pH has declined faster than from the CO_2_-effect alone^8^. Because of the complex combinations of processes that modify pH in coastal seas, it is not straightforward to predict pH development for the coming decades. Sensitivity experiments do nevertheless indicate that if future CO_2_ emissions are not abated, it is unlikely that other processes in the Baltic Sea can completely counteract the acidification trend induced by the atmospheric CO_2_-increase in the long run^14^.

The main objectives of this study are: (1) to recap the functioning and driving factors of long-term trends and short-term variations of the acid–base system, (2) to discuss possible consequences for marine organisms, and (3) to discuss implications for monitoring and management.

## Results

The past, present-day, and potential future development of acidification in the Baltic Sea is addressed by means of model simulations using reconstructed and observed model forcing, as well as different future CO_2_ emission and climate change scenarios combined with nutrient load scenarios. The model simulations are described in “Model simulations” section.

### Trends of acidification

**Figure 1:**
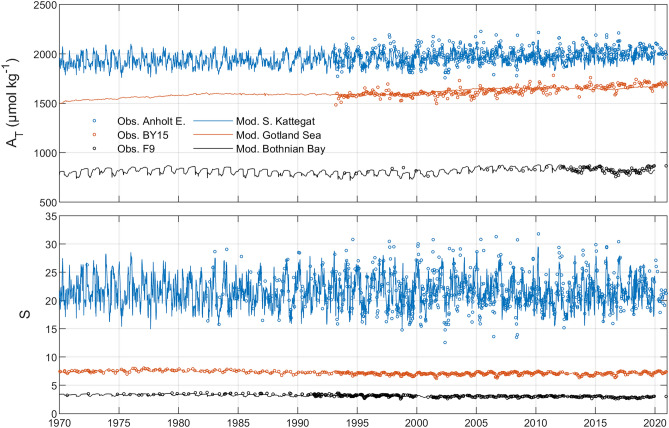
Simulated (lines) and observed (circles) surface water A_T_ and salinity (S) in three model sub-basins and representative monitoring stations (cf. Fig. S1, supporting information).

Observations and model simulations for A_T_ and S agreed well over a 50-year period (Fig. 1). The three stations represent the salinity gradient from the low-saline northern Baltic Sea towards the saline North Sea, which was paralleled by an increasing A_T_ gradient. Salinity variations in the southern Kattegat were more dynamic compared to the two other basins, which also had effects on the A_T_ variability. A_T_ observations from before 1993 have been reported in previous studies, but these observations have been rendered unreliable more recently by the institute responsible for the monitoring (Swedish Meteorological and Hydrological Institute, SMHI) in line with data analysis of long-term A_T_ data^7^.

**Figure 2:**
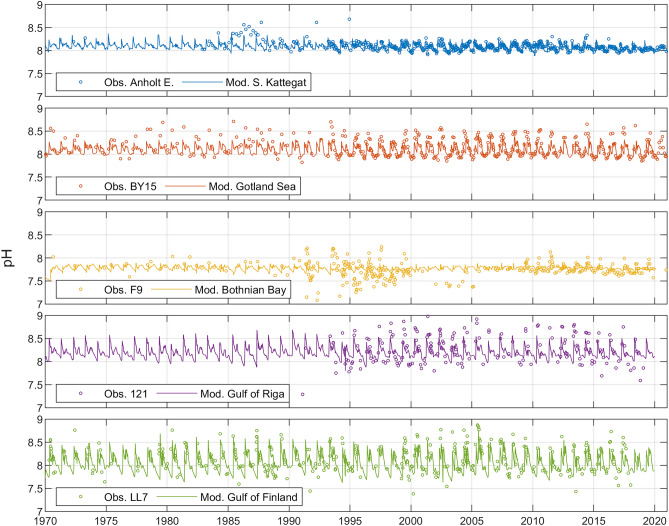
Simulated (lines) and observed (circles) time series of surface water pH in five model sub-basins and representative monitoring stations (cf. Fig. S1, supporting information).

In contrast to salinity and A_T_, pH exhibited pronounced seasonal variations in the different basins with a tendency for larger excursions in the basins with lower A_T_ (Fig. 2). The exception is the oligotrophic Bothnian Bay where low primary production dampened the pH seasonal variation despite low buffering capacity (Fig. 1).

The range of observed pH from the F9 station (Bothnian Bay) from the early 1990s until 2005 is extremely large compared to the observed ranges in the periods 1970–1990 and 2006–2019, respectively (Fig. 2). Further, observed pH data from the Kattegat in the period 1985–1988 deviate from observations in other years. The reasons for these deviations are not known, but it is likely that these data could be corrupted. The average pH at station F9 was considerably lower than pH observed at stations in the other basins. Statistics for observed and modelled A_T_ and pH, respectively, at different stations and corresponding sub-basins over the period 1993–2019 are summarized in Table 1 (pH observations from before 2006 at station F9 were however excluded in this comparison because of the abovementioned uncertainties).

**Table 1:** Comparison between observed and modelled surface water A_T_ (µmol kg^-1^) and pH (mean ± standard deviation) over the time period 1993–2019, except for pH at F9/Bothnian Bay that only include the period 2006–2019 because of questionable data before 2006 (see text).

	Anholt E./S. Kattegat	BY15/Gotland Sea	F9/Bothnian Bay	121/Gulf of Riga	LL7/Gulf of Finland
Obs. A_T_	1986 ± 80	1624 ± 47	826 ± 26		
Mod. A_T_	1965 ± 59	1631 ± 29	814 ± 33	2236 ± 70	1483 ± 32
Obs. pH	8.08 ± 0.08	8.15 ± 0.20	7.78 ± 0.10	8.30 ± 0.30	8.17 ± 0.31
Mod. pH	8.04 ± 0.06	8.07 ± 0.09	7.77 ± 0.03	8.18 ± 0.10	8.02 ± 0.16

Note that not all basins had sufficient observational data to determine A_T_.

### Future projections

**Figure 3:**
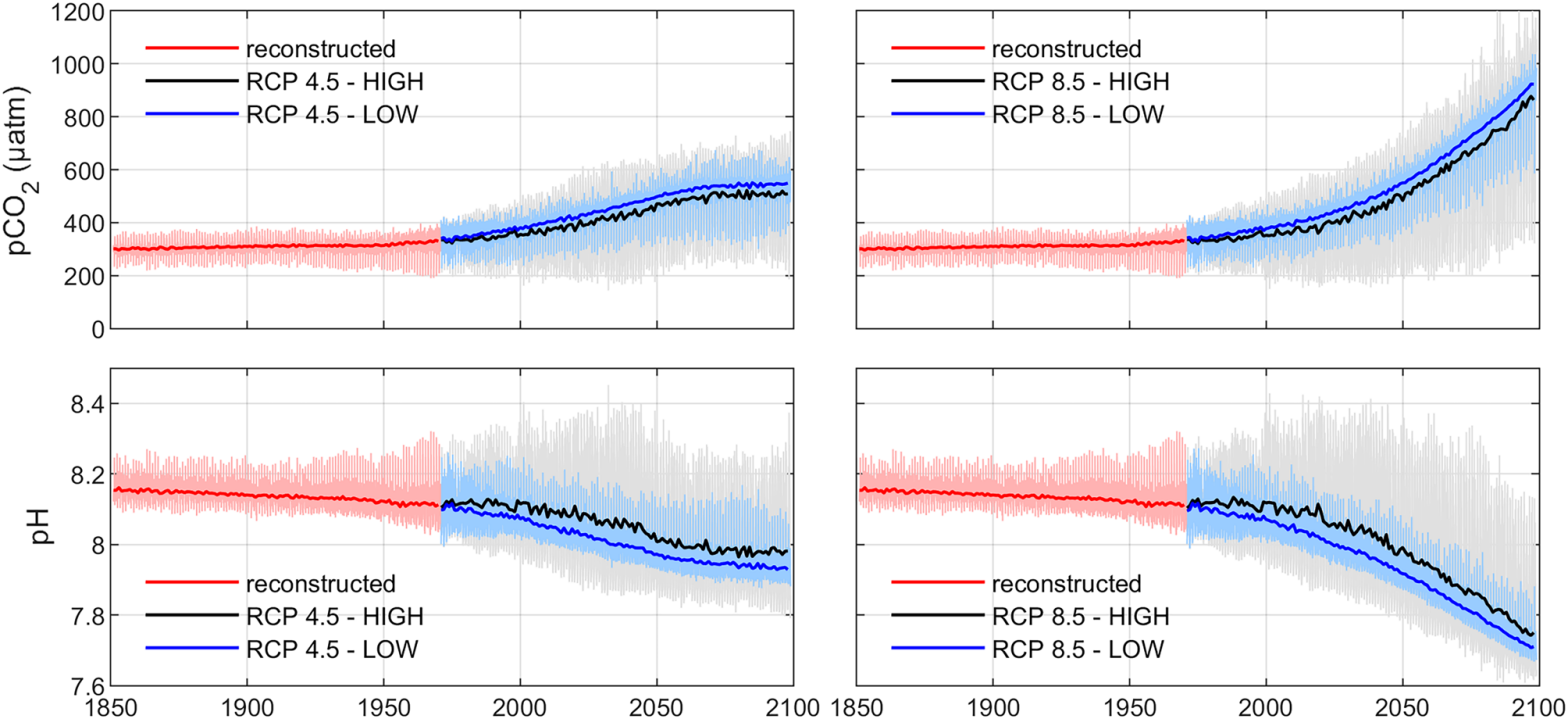
Simulated surface water pCO_2_ (upper panel) and pH (lower panel) in the Gotland Sea, using reconstructed forcing for the period 1851–1970 and climate change scenarios combined with nutrient load scenarios for the period 1971–2098 (cf. Appendix A2, supporting information). Thin pale lines indicate short-term variations while thick bright lines indicate annual means.

Model simulations were used to investigate differences between past, present, and possible future conditions with regards to inorganic carbon system parameters in the Baltic Sea (Fig. 3). Atmospheric CO_2_ levels and meteorological forcing according either to the RCP 4.5 or the RCP 8.5 emission scenario were used for simulations of the potential future development (see “Model simulations” section). These emission scenarios were combined with two alternative nutrient load scenarios; either with loads based on the maximum allowable loads according to the Baltic Sea Action Plan (LOW), or with loads based on the extremely high inputs observed in the 1980’s (HIGH) (see “Model simulations” section).

The two different climate scenarios resulted in large differences in pCO_2_ and pH. Whereas pCO_2_ would reach levels around 500 µatm under the intermediate climate scenario (RCP 4.5) by the end of the century, pCO_2_ would approach 1000 µatm under the business-as-usual scenario (RCP 8.5). Noteworthy is the acceleration of acidification under RCP 8.5 starting already before 2050. Similarly, pH levels around 8 would be expected by 2100 under the RCP 4.5 scenario, whereas pH would drop as low as 7.7–7.8 under the RCP 8.5 scenario. Compared to the climate scenarios, the effect of nutrient loads in the scenarios (HIGH and LOW, respectively) was relatively smaller, yielding a pCO_2_ increase around 30 µatm under both climate scenarios if nutrient loads are kept low. Similarly, the low nutrient scenario would lead to lower pH values (around 0.02 lower) under both climate scenarios. Hence, curbing CO_2_ emissions is more important than managing nutrient loads with regard to acidification in the Baltic Sea. However, nutrient load reduction leads to a decrease in the seasonal amplitude of the surface water pH and associated extremes (i.e., annual minimum and maximum values).

**Figure 4:**
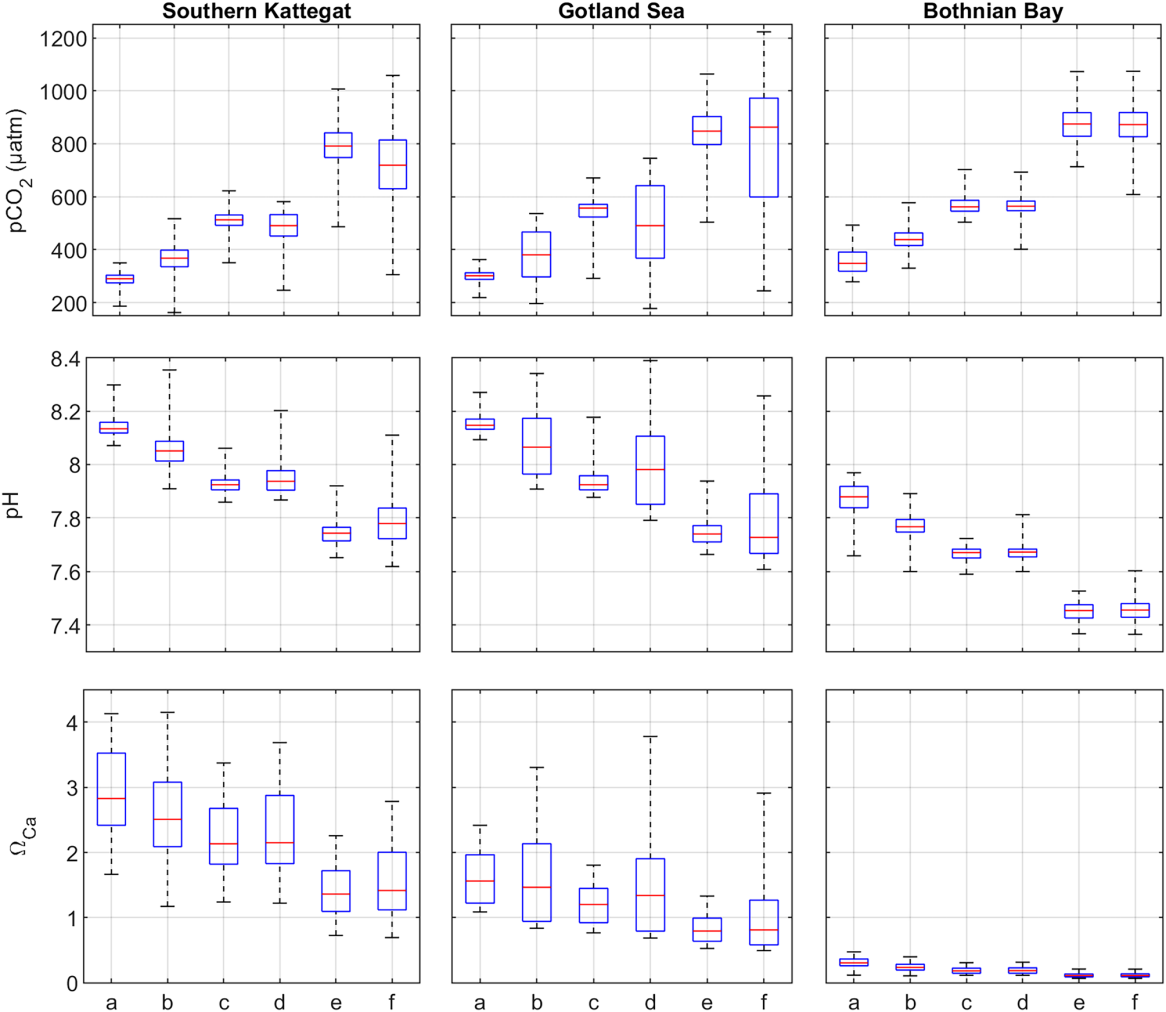
Simulated distributions of surface water pCO_2_, pH, and Ω_Ca_ over the 20-year periods indicated in Table 2. Red lines mark the median values of the various datasets, whiskers indicate the span between minimum and maximum values, boxes are drawn from the median of the lower half of the datasets to the median of the upper half of the datasets.

Model simulations highlight substantial changes of the inorganic carbon system over the last 150 years across all basins in the Baltic Sea, and these acidification trends are expected to continue in the future, albeit with variable outcome depending on how CO_2_ and nutrient emissions are controlled (Fig. 4). Today’s levels of pCO_2_ have already increased by about 89 µatm in the Kattegat, 93 µatm in the Gotland Sea and 85 µatm in the Bothnian Bay compared to the pre-industrial period. In parallel, pH levels have decreased by 0.09 in the Kattegat, 0.09 in the Gotland Sea and 0.10 in the Bothnian Bay. Similar ranges of change are expected by the end of the century, provided that CO_2_ emissions are curbed, but if CO_2_ emissions continue to rise without any significant reduction measures, the changes observed so far are going to be more than doubled. This has severe consequences for the solubility of calcium carbonate minerals, as shown for the saturation state of calcite (Ω_Ca_) (Fig. 4). In the well-buffered Kattegat, calcite will remain saturated under RCP 4.5, but Ω_Ca_ may drop occasionally below 1 under RCP 8.5. In the Gotland Sea, calcite will largely remain saturated under RCP 4.5 although periods of Ω_Ca_ < 1 occur (typically in winter), but under RCP 8.5 the Gotland Sea will mainly be undersaturated with calcite and Ω_Ca_ > 1 predominantly occurring during algae blooms. In the Bothnian Bay, saturation states are already low due to poor buffering and these undersaturation conditions will intensify in the future. The nutrient scenarios have a moderate effect on the median levels of the inorganic carbon system variables. However, nutrient reductions have a drastic effect on reducing the variability in these parameters as indicated by the ranges of the boxes and whiskers (Fig. 4).

**Table 2:** Time periods and forcing data used for simulating inorganic carbon system variables (cf. Figure 4).

Notation	Description	Time period considered	Atmospheric pCO_2_ and meteorological data	Nutrient supply
a	Pre-industrial	1851–1870	Reconstructed	Reconstructed
b	Present-day	2000–2019	Observed	Observed
c	Moderate CO_2_ increase, low nutrient loads	2079–2098	RCP 4.5	LOW
d	Moderate CO_2_ increase, high nutrient loads	2079–2098	RCP 4.5	HIGH
e	Large CO_2_ increase, low nutrient loads	2079–2098	RCP 8.5	LOW
f	Large CO_2_ increase, high nutrient loads	2079–2098	RCP 8.5	HIGH

**Figure 5:**
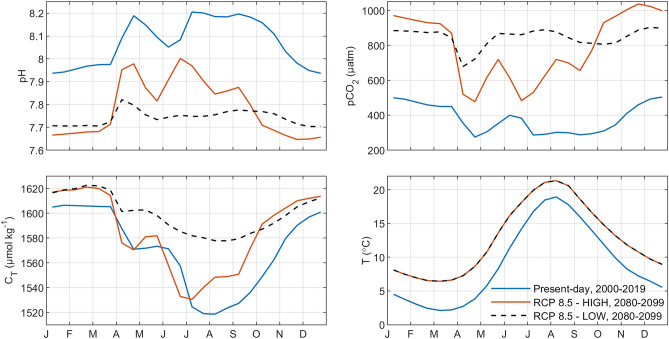
Simulated average seasonal variations of surface water pH, pCO_2_, C_T_, and T in the Gotland Sea, comparing different time-periods and nutrient load scenarios (cf. Table 2).

The changing variability among scenarios was mostly expressed in changing seasonal patterns (Fig. 5). The RCP 8.5 with HIGH nutrient loads corresponded mainly to a displacement of the present seasonal variation in pH by approximately 0.3 units. For pCO_2_, the seasonal variation was larger under RCP 8.5 HIGH, varying from 600 to 1000 µatm compared to a present-day range of around 200 µatm (300–500 µatm). The dissolved inorganic carbon concentration (C_T_) decreased under the RCP 8.5 scenarios because of decreasing salinity and A_T_—lower salinity means a lower proportion of ocean water, which in turn results in lower A_T_ (not shown).

## Discussion

CO_2_ absorbed from the atmosphere reacts with water and equilibrates with bicarbonate and carbonate. For that reason, the time it takes to equilibrate surface layer C_T_ with atmospheric CO_2_ is long compared to equilibration times for other gases that do not react with water. The difference in equilibration times for CO_2_ and other gases can be expressed by the Revelle factor, which is defined as the ratio of instantaneous change in pCO_2_ to change in C_T_^2^. This factor can be interpreted as a measure of the ocean’s capacity to buffer changes in CO_2_ due to absorption of atmospheric CO_2_^17^, but indirectly also gives an indication of pH sensitivity to C_T_ changes at constant A_T_—high Revelle factor means low buffering capacity. The present-day Revelle factor of Baltic Sea surface water is typically in a range ~ 20–25, which is high compared to the surface ocean of ~ 10 on average^2^. This means that the Baltic Sea can be expected to experience acidification effects more pronounced than the open ocean.

Observed A_T_ concentrations and pH levels in different areas of the Baltic Sea can be reproduced by the BALTSEM model, as demonstrated in Figs. 1 and 2. However, simulated seasonal pH variations tend to be underestimated compared to observed seasonal variations, and the reason for this is that the CO_2_ uptake by autotrophs is underestimated in the model^14^. The fact that the model is able to reasonably well capture observed past and present-day conditions implies that it is also a viable tool for sensitivity experiments and future scenario simulations: BALTSEM has been used in numerous studies focused on the sensitivity and possible future development related to both climate change and nutrient loads change^14, 18–22^.

The model simulations demonstrate that atmospheric CO_2_ is the dominant factor driving long-term acidification trends and shifting the baselines, or typical values, of pCO_2_, pH, and saturation levels of calcium carbonate. Surface water pCO_2_ tends to follow the long-term atmospheric CO_2_ development (see Figure S2) with considerably higher pCO_2_ in the RCP 8.5 scenario than in the RCP 4.5 scenario (Fig. 3). The long-term development of pH mirrors that of pCO_2_. The considerably higher productivity in the scenarios with HIGH nutrient loads results in amplified seasonal variations of pCO_2_ and pH compared to the LOW nutrient load scenarios because of a larger CO_2_ uptake by autotrophs in spring and summer, and consequently a larger CO_2_ release during mineralization of excess organic matter. These results support earlier studies on the response of inorganic carbon system parameters to climate change and changes in nutrient loads^14, 23^.

Annual mean pCO_2_ is slightly lower in the HIGH load scenarios than in the LOW load scenarios, and the annual mean pH is correspondingly slightly higher in the HIGH load scenarios. These differences depend on one hand on the more efficient carbon removal (by autotrophic uptake and subsequent sedimentation and burial) in the HIGH scenarios but also to a smaller degree on slightly higher A_T_ concentrations in the HIGH scenarios (not shown). The higher A_T_ concentrations in the eutrophic scenarios is mainly linked to nitrogen cycling. The external supply of nitrate is higher in the HIGH scenarios, but on the other hand, the removal of nitrate by denitrification is also higher because of poor oxygen conditions in the deep water. Removal of externally supplied nitrate is a net A_T_ source^14^. It is however important to note that the lowest pH values on a seasonal scale occur in the HIGH nutrient load scenarios as a result of the enhanced seasonal carbon cycling during eutrophic conditions (Figs. 3 and 4).

Simulated surface water pCO_2_ increased by more than 100 µatm over the past 150 years (Fig. 4), matching the development of atmospheric CO_2_ (Fig. S2). As a result, pH decreased by approximately 0.1. Nutrient loads to the Baltic Sea increased substantially over the twentieth century^24^ resulting in enhanced primary production and thus enhanced seasonal variations of inorganic carbon system parameters. For that reason, the seasonal ranges of pCO_2_, pH, and Ω_Ca_ have increased in the southern Kattegat and the Gotland Sea over time. While the Bothnian Bay is influenced by eutrophication effects (e.g. increased heterotrophy and decreased water transparency), these changes are in contrast to other areas of the Baltic Sea not reflected in a strongly increased autotrophy. This means that the seasonal ranges of present-day inorganic carbon system parameters are similar to seasonal ranges during pre-industrial conditions (Fig. 4).

It is not only the atmospheric pCO_2_ that differs between the RCP 4.5 and RCP 8.5 scenarios, but also runoff and meteorological forcing. As a consequence, there are differences between these scenarios in terms of e.g. A_T_, temperature, salinity, stratification, and water exchange between the Baltic Sea and the North Sea—factors that also have an impact on the inorganic carbon system. This adds complexity when interpreting the results from the different future scenario simulations. We are not going to delve deeply into these comparatively small differences between different simulations; the inorganic carbon system’s sensitivity to changes in e.g. runoff, A_T_ and C_T_ supplies, and air temperature, has previously been described in detail^14^.

The seasonality of inorganic carbon system parameters is expected to change in the future as a result of the gradual warming of Baltic Sea waters. The differences in average levels of pH and pCO_2_ between present-day and potential future conditions (Fig. 5) depend largely on the atmospheric pCO_2_ level. In the RCP 8.5 scenario, the surface water becomes 2–3 °C warmer towards the end of the twenty-first century compared to present-day conditions. This has the effect that plankton blooms can occur several weeks earlier compared to today, since growth rates of autotrophs depend on temperature (among other factors, such as light and nutrient conditions).

All four parameters of the inorganic carbon system (pH, pCO_2_, A_T_, and C_T_) are nowadays straightforward to measure, and Standard Operating Procedures (SOPs) exist^25, 26^. However, precise measurements of pH remain challenging in the brackish waters of the Baltic Sea because of shortcomings of the potentiometric measurements that are generally used. Recent advances in technology regarding spectrophotometric pH measurements suggest that the problems of accurate and precise pH measurements in brackish waters as well as waters strongly influenced by dissolved organic matter could be overcome, thus allowing measurements that are sufficiently precise to monitor long-term acidification trends^27–30^.

Observations of both A_T_ and pH in the Baltic Sea date back to the early twentieth century, but these parameters have not been monitored consistently across the Baltic Sea. Precision and quality of the data also vary considerably over time. In particular, all A_T_ observations from before 1993 are now deemed unreliable by the institute responsible for the measurements. Furthermore, pH data from some stations appear to be highly questionable at certain periods in time (e.g., pH data from the F9 station in the period 1990–2006, see “Trends of acidification” section).

A_T_ and pH are regularly monitored by SMHI at open water stations, but they are not regular monitoring parameters in most of the other Baltic Sea coastal states. In addition, pCO_2_ measurements are available from ferry box systems on ships-of-opportunity, covering the major Baltic Sea basins^31, 32^. As a consequence of the temporal and spatial limitations of high quality data, assessment of acidification trends in the Baltic Sea is currently largely confined to open water stations and a limited number of coastal stations where data are available and studies have been made^8^.

The complex situation in the Baltic Sea, with large differences between sub-basins and between open-water and coastal areas, gives a strong incentive to improve the temporal and spatial coverage of acidification monitoring. This would on one hand broaden the understanding of current acidification trends, but in addition also improve the Baltic Sea models’ capacity to assess future changes.

Monitoring of inorganic carbon system parameters also provides an added value as an indicator of eutrophication. Phytoplankton production and mineralization of organic carbon are, as described above, the major drivers of seasonal surface water pCO_2_ variations. If phytoplankton assimilate carbon, nitrogen, and phosphorus according to fixed ratios (Redfield), that would imply that the CO_2_ assimilation, the surface-to-deep water export of organic carbon, and the associated deep water oxygen demand could be addressed based on knowledge of inorganic nitrogen and phosphorus inventories. Recent studies have, however, indicated considerable variations in terms of the carbon, nitrogen, and phosphorus ratios of planktonic uptake in the Baltic Sea^33–37^.

Measurements of inorganic carbon system parameters are thus on one hand necessary to monitor acidification in the Baltic Sea. But on the other hand, an added value of such measurements is that they can also be used to quantify primary production directly based on carbon cycling, and in extension improve the understanding of the linkage between primary production and deep water oxygen demand. Measurements of surface water pCO_2_ provide the most robust estimate of long-term eutrophication trends since they do not rely on assumptions about the stoichiometry of autotrophs^38, 39^.

If CO_2_ emissions were to be reduced sufficiently to fulfill the goal of the Paris agreement, i.e., to limit warming to below 2 °C (and preferably below 1.5 °C) compared to pre-industrial levels, this would mean that the atmospheric CO_2_ level would culminate and start to decrease before the end of the twenty-first century. Decreasing atmospheric CO_2_ would in turn drive positive pH trends in ocean surface waters globally. In the worst-case scenario (i.e., RCP 8.5), pH could in contrast decrease by 0.3 units or more by year 2100 compared to present-day levels.

Ocean acidification leads to reduced calcification of shell-forming organisms because of the gradual decline in saturation levels of calcium carbonate minerals, and in addition affects a range of other physiological processes—in particular processes related to cellular ion regulation^40^. The large seasonal variations of pH and pCO_2_ observed in Baltic Sea surface waters indicate that organisms there are adapted to cope with wide ranges of pH and pCO_2_ in their environment. Increasing atmospheric CO_2_ is gradually going to shift the natural ranges of pH and pCO_2_ that are observed in different areas of the Baltic Sea, but it is likely that long-term adaptations to increasing pCO_2_ and decreasing pH occur in the Baltic Sea on both species and community levels^41, 42^. Observed responses to acidification of 1. plankton communities, 2. benthic communities, and 3. fish in the Baltic Sea are broadly summarized below with a few examples highlighting key species.

1. Plankton communities in the Baltic Sea appear to be tolerant to pCO_2_ levels in a range 1000–1400 µatm^42, 43^, which is above the estimated worst case pCO_2_ increase over the twenty-first century (Fig. 3). Further, phytoplankton and nitrogen fixing cyanobacteria show mainly subtle responses to acidification^44–48^. It has however been suggested that high pCO_2_ could influence the plankton community composition by supporting picoeukaryotic primary producers and small sized microzooplankton and also decreasing the diversity of the microzooplankton community in summer^46, 49^. Experiments indicate that the Baltic Sea mesozooplankton community is tolerant to acidification^50^, whereas copepods could be either tolerant or negatively affected at high pCO_2_ levels^51, 52^.
2. Experiments indicate that all life forms of the bivalve *Limecola balthica* react negatively to acidification that causes both a decreased growth rate and survival of the larval stage as well as increased metabolic rates and energy demand of the adult stage^53^. Adult stages of the bivalves *Mytilus edulis* and *Arctica islandica*, on the other hand, appear more tolerant to high pCO_2_ levels^54–56^. Negative impact of acidification on adult bivalve growth is mainly related to an increasing energy demand for maintaining the physiological balance^53, 57^. It has thus been suggested that a sufficient food supply could compensate for the negative effects of increasing pCO_2_^55, 58^. Adult barnacles from habitats with high pCO_2_ fluctuations have been shown to be more tolerant to high pCO_2_ levels than barnacles from habitats with comparatively stable pCO_2_ levels^58^. The more sensitive populations have further been shown to be less capable to adapt to long-term elevated pCO_2_, which significantly effects reproduction^59^. Growth of the seagrass *Zostera marina* appears to be weakly positively influenced by increasing pCO_2_^60^, whereas responses of the macroalgae *Fucus vesiculosus* differ between studies—from negative to weakly positive^60, 61^. Red algae seem to respond positively to acidification, whereas for green algae both positive and negative responses have been observed^60^.
3. Baltic Sea cod could potentially respond negatively to acidification, although for cod larvae contrasting results (either tolerant or strongly negatively impacted by high pCO_2_) have been reported from different experiments^41, 62^. Experiments further indicate that acidification may lead to a decreased larval growth of herring^63^. Additionally, potential effects of acidification on the food web could negatively impact fish populations by a decreased food availability.

It should be noted that information on species’ responses to acidification is sometimes based on relatively short-term experiments, and there are major knowledge gaps both regarding effects of long-term exposure^59^ and the capacity of species and ecosystems to adapt^64^. Recent studies have emphasized the need to account for effects of multiple stressors (e.g. acidification, warming, de-oxygenation) on communities as well as habitat structure and complexity, rather than the response of a single species to a single factor^64–68^.

To summarize, the current knowledge of how acidification could affect organisms and ecosystems in the Baltic Sea has been reviewed in detail^69^. Assuming pCO_2_ ≤ 1300 µatm and pH decrease ≤ 0.4, some main tendencies have been identified: (1) acidification effects varied from strongly positive to strongly negative depending on species, but with negative effects more common in macrobenthos and fish than in plankton, (2) acidification could drive planktonic systems toward the microbial loop, (3) this could in turn also impact zooplankton and fish because of a reduced food availability, (4) negative consequences of acidification can be partly amended by increased food availability, and (5) there are still knowledge gaps regarding the ecological and evolutionary capacity of ecosystems to cope with acidification in combination with other ecosystem pressures, such as climate change, eutrophication, overfishing, and hazardous substances.

It is clear that there are no universal thresholds for acidification parameters that apply to all organisms. The difficulty to assign thresholds has implications for management because of the uncertainties in what to expect regarding possible ecosystem changes. However, even if it is uncertain exactly how acidification will affect marine organisms, our simulations indicate large changes of the inorganic carbon system, which means it is important to assess and evaluate the change as it happens. Furthermore, long-term acidification trends in Baltic Sea waters depend on global CO_2_ emissions. This means that in contrast to nutrient-driven eutrophication, which can be managed by means of regional measures, it is difficult to set future goals in terms of acidification management. It is nevertheless important to continuously monitor the development of acidification and also to improve the knowledge of possible consequences for marine organisms and ecosystems. The multi-stressor approach is mentioned in this context^69^, meaning that the combined effect of acidification and other ecosystem pressures is a bigger threat than effects of the individual stressors. If it is not possible to manage acidification, one could arguably aim to minimize effects of other stressors in the habitats that are particularly vulnerable to acidification effects.

## Methods

### The marine inorganic carbon system

The inorganic carbon system is defined by four analytically quantifiable parameters: dissolved inorganic carbon (C_T_), total alkalinity (A_T_), partial pressure of CO_2_ (pCO_2_), and pH. The system can be determined if any two of these parameters are known, together with values of acid–base dissociation constants and total concentrations of other ions, notably borate^25^. The inorganic carbon system in coastal seas is influenced by processes in their catchments, exchange with the open ocean, air-sea exchange, as well as physical and biogeochemical processes in water and sediments. Specific anomalies and peculiarities of the acid–base balance in the Baltic Sea, such as dissociation constants in brackish water, contributions from organic alkalinity, anomalies of borate alkalinity, long-term A_T_ changes, as well as the effects of production and mineralization have been reviewed^70^.

### Data

Marine monitoring data were extracted from the ICES oceanographic database (ICES Data Set on Ocean Hydrography, the International Council for the Exploration of the Sea, Copenhagen, http://ocean.ices.dk/Helcom/) and the Swedish Ocean Archive (SHARK) database provided by the Swedish Meteorological and Hydrological Institute (SMHI; https://sharkweb.smhi.se/hamta-data/). Atmospheric CO_2_ data were downloaded from the RCP database (Representative Concentration Pathways database, version 2.0.5, https://tntcat.iiasa.ac.at/RcpDb/dsd?Action=htmlpage&page=download).

### Model simulations

For the model simulations we used BALTSEM, a coupled hydrodynamic-biogeochemical model for the Baltic Sea^24^. The model simulates salinity, temperature, dissolved oxygen, dissolved inorganic and organic nutrients and carbon, detrital nutrients and carbon, three autotroph groups, one bulk state variable for heterotrophs, the marine inorganic carbon system parameters (C_T_, A_T_, pCO_2_, and pH), and saturation levels of calcite and aragonite (Ω_Ca_ and Ω_Ar_). BALTSEM has been described and validated in numerous publications. A brief overview with relevant references is provided in Appendix A1 (supporting information).

Model output from three different time periods were compared. These periods represent pre-industrial conditions (model output from 1851 to 1870), contemporary conditions (model output from 2000 to 2019), and possible future conditions (model output from 2079 to 2098). For the future scenario simulations, we used atmospheric CO_2_ levels and meteorological forcing according either to the RCP 4.5 or the RCP 8.5 emission scenario^71^.

These two scenarios were then combined with two alternative nutrient load scenarios. In the nutrient load scenario called LOW we used nutrient loads based on maximum allowable inputs according to the Baltic Sea Action Plan^72^. The total nutrient supply from land and atmosphere in the LOW scenario amounts to 766 kton N y^−1^ and 20 kton P y^−1^, respectively. These loads were set to be constant throughout the model runs. In the scenario called HIGH, the nutrient loads were based on observed inputs to the Baltic Sea in the 1980’s (the period with highest recorded loads). The total nutrient supply from land and atmosphere in the HIGH scenario amounts to 1281 kton N y^−1^ and 66 kton P y^−1^, respectively. Again, the loads were set to be constant throughout the model runs.

Longer time-series of riverine C_T_ and A_T_ concentrations were not available for some of the major Baltic Sea rivers, particularly in the southeastern catchments. For that reason, we have assumed constant riverine A_T_ and C_T_ concentrations in our simulations. The concentrations were held constant over time, but vary among rivers entering different sub-basins of the system. These average concentrations were based on calibrated A_T_ and C_T_ supplies^20, 21^.

### Supplementary Information

Supplementary Information.

Supplementary Figures.

## Data Availability

The datasets generated during and/or analyzed during the current study are available from the corresponding author on reasonable request.
