# Supplementary material for: Causes and consequences of acidification in the Baltic Sea: implications for monitoring and management
Source: Sci Rep. 2023 Sep 28;13:16322. doi: 10.1038/s41598-023-43596-8 (PMC10539381; doi:10.1038/s41598-023-43596-8)
Supplement: Supplementary file 1 — Supplementary Information. [file 41598_2023_43596_MOESM1_ESM.pdf]

Supplementary information: ***Causes and consequences of acidification in the Baltic Sea: implications for monitoring and management***

Erik Gustafsson[*,1], Jacob Carstensen[2], Vivi Fleming[3], Bo G. Gustafsson[1,4], Laura Hoikkala[3], Gregor Rehder[5]

[1] Baltic Nest Institute, Baltic Sea Centre, Stockholm University, Stockholm, Sweden

[2] Department of Ecoscience, Aarhus University, Roskilde, Denmark

[3] Marine and freshwater solutions, Finnish Environment Institute, Helsinki, Finland

[4] Tvärminne Zoological Station, University of Helsinki, Hanko, Finland

[5] Leibniz Institute for Baltic Sea Research Warnemünde (IOW), Rostock, Germany

[*] Correspondence to: Erik Gustafsson (erik.gustafsson@su.se)

Appendix

A1. Model description

The hydrodynamic module is forced by weather data with a 3-hour resolution (wind, temperature, humidity, cloudiness, air pressure, and precipitation), daily sea level in the Kattegat, daily vertical gradients of salinity and temperature in Skagerrak (the outer boundary), and monthly river runoff. The biogeochemical module additionally needs monthly land loads and atmospheric depositions of nutrients, carbon, and A_T, as well as daily concentrations of these parameters in the Skagerrak. Detailed descriptions and model validations are available[1, 2, 3].

A2. Forcing data

A2.1. Reconstructed data covering the period 1851-2006

Atmospheric forcing, river runoff, sea-level forcing, land loads, atmospheric depositions, and also boundary conditions in the Skagerrak have been described in detail[1].

A2.2. Observed nutrient loads and actual weather data, covering the period 1970-2019

- Atmospheric forcing is constructed from the following datasets that were provided by the SMHI: RCA-ERA40 (1970-2006), Hirlam-Mesan (2007-2015), and Arome-Mesan (2016-2019).
- River runoff, land loads, and atmospheric depositions are based on Pollution Load Compilation data (PLC)[4] and other sources[1].
- The Kattegat water level as well as boundary conditions in the Skagerrak are based on data provided by the SMHI[24].

A2.3. Future scenarios covering the period 1971-2098

The regional climate data sets and climate change projections RCP 4.5 and RCP 8.5 used to construct forcing data have been described in detail[5].

References

1. Gustafsson, B. G. *et al.* Reconstructing the Development of Baltic Sea Eutrophication 1850–2006. *AMBIO* **41**, 534–548 (2012).
2. Savchuk, O. P., Eilola, K., Gustafsson, B. G., Medina, M. R. & Ruoho-Airola, T. *Long-term reconstruction of nutrient loads to the Baltic Sea, 1850-2006* (2012).
3. Gustafsson, E., Savchuk, O. P., Gustafsson, B. G. & Müller-Karulis, B. Key processes in the coupled carbon, nitrogen, and phosphorus cycling of the Baltic Sea. *Biogeochemistry* **134**, 301–317 (2017).
4. HELCOM. *HELCOM Baltic Sea Action Plan – 2021 update* (2021). Available at http://helcom.fi.
5. Saraiva, S. *et al.* Baltic Sea ecosystem response to various nutrient load scenarios in present and future climates. *Clim Dyn* **52**, 3369–3387 (2019).
