# Supplementary material for: Causes and consequences of acidification in the Baltic Sea: implications for monitoring and management
Source: Sci Rep. 2023 Sep 28;13:16322. doi: 10.1038/s41598-023-43596-8 (PMC10539381; doi:10.1038/s41598-023-43596-8)
Supplement: Supplementary file 2 — Supplementary Figures. [file 41598_2023_43596_MOESM2_ESM.pdf]

Supporting information: ***Causes and consequences of acidification in the Baltic Sea – implications for monitoring and management***

Erik Gustafsson[*,1], Jacob Carstensen[2], Vivi Fleming[3], Bo G. Gustafsson[1,4], Laura Hoikkala[3], Gregor Rehder[5]

[1] Baltic Nest Institute, Baltic Sea Centre, Stockholm University, Stockholm, Sweden

[2] Department of Ecoscience, Aarhus University, Roskilde, Denmark

[3] Marine and freshwater solutions, Finnish Environment Institute, Helsinki, Finland

[4] Tvärminne Zoological Station, University of Helsinki, Hanko, Finland

[5] Leibniz Institute for Baltic Sea Research Warnemünde (IOW), Rostock, Germany

[*] Correspondence to: Erik Gustafsson (erik.gustafsson@su.se)

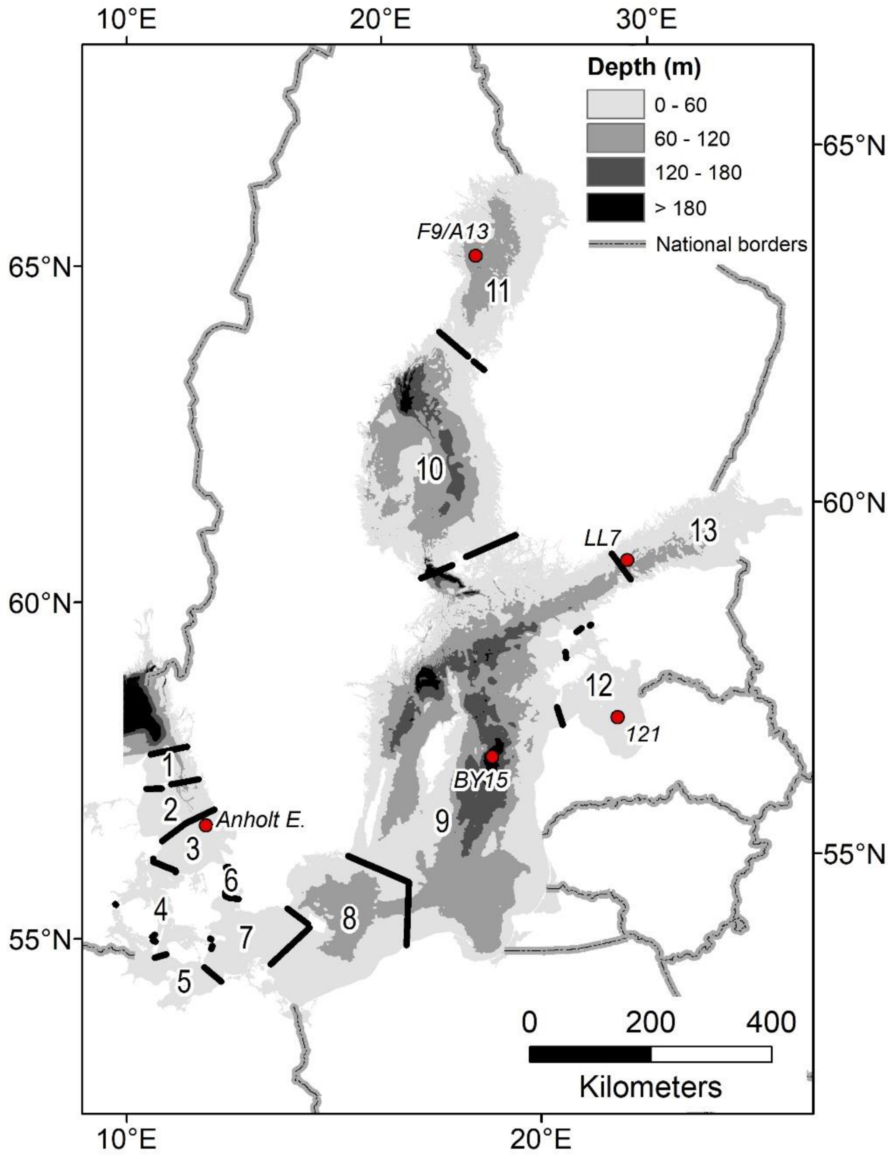

Figure S1. Map over the Baltic Sea, including a few selected monitoring stations as well as the thirteen sub-basins of the BALTSEM model: 1. Northern Kattegat (NK), 2. Central Kattegat (CK), 3. Southern Kattegat (SK), 4. Samsø Belt (SB), 5. Fehmarn Belt (FB), 6. Öresund (OS), 7. Arkona Basin (AR), 8. Bornholm Basin (BN), 9. Gotland Sea (GS), 10. Bothnian Sea (BS), 11. Bothnian Bay (BB), 12. Gulf of Riga (GR), 13. Gulf of Finland (GF).

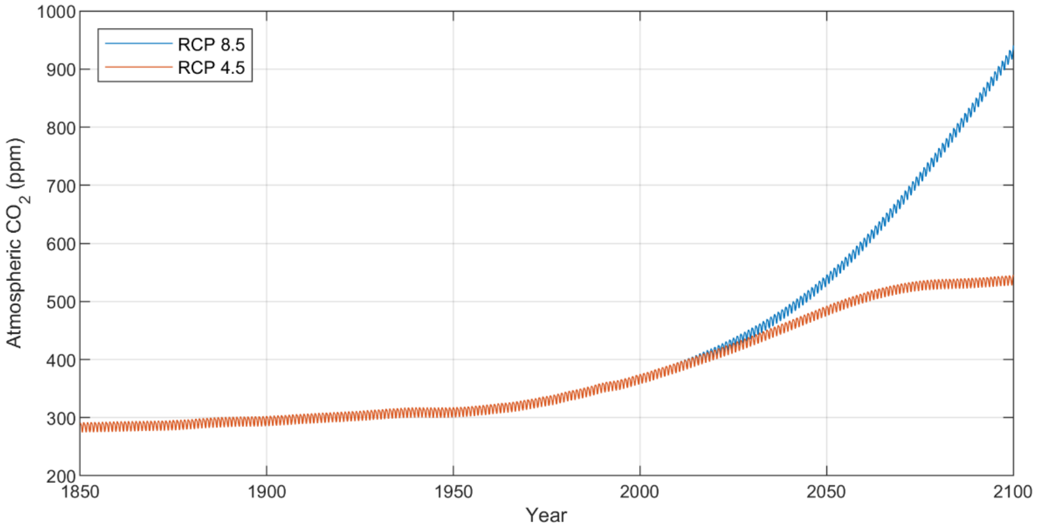

Figure S2. Past and possible future atmospheric CO_2 level according to the RCP 4.5 and RCP 8.5 scenarios respectively.
